# HIV infection among men who have sex with men in Israel: a 35-year epidemiological and clinical overview, 1981–2015

**DOI:** 10.1186/s12889-019-7000-1

**Published:** 2019-06-13

**Authors:** D. Chemtob, Z. Mor, N. Harel, N. Averick

**Affiliations:** 1 Department of Tuberculosis & AIDS, Ministry of Health, Jerusalem, Israel; 2 Faculty of Medicine, Braun School of Public Health & Community Medicine, Hebrew University-Hadassah Medical School, Jerusalem, Israel; 3 Tel Aviv Department of Health, Ministry of Health, Tel Aviv, Israel; 4 School of Health Sciences, Ashkelon Academic College, Ashkelon, Israel

**Keywords:** Acquired immunodeficiency syndrome – AIDS, Human immunodeficiency virus – HIV, Men who have sex with men – MSM, Injecting drug users - IDU, Israel, Gay men

## Abstract

**Background:**

This study is the first to describe major epidemiological trends and clinical characteristics among Israeli men who have sex with men (MSM), who are at a higher risk for HIV infection.

**Methods:**

This retrospective study includes all individuals reported to the Israeli Ministry of Health with HIV and self-identified as MSM between 1981 and 2015. The incidence rates of HIV infection and AIDS-defining diseases were analyzed and Kaplan-Meier survival estimates were calculated from time of HIV infection notification to AIDS diagnosis and death across three consecutive periods representing antiretroviral treatment availability.

**Results:**

The trend of increase in HIV incidence is similar to Western Europe, although Israeli rates are lower. Of 2052 HIV/AIDS Israeli MSM diagnosed during the follow-up, 296 (14.6%) developed AIDS. MSM constitute 28.4% of all HIV/AIDS cases and 41.5% of cases among men. Average times from HIV-notification until AIDS diagnosis were 15.5 [14.0–16.9], 16.0 [15.5–16.4], and 6.7 [6.7–6.8] years, within 1981–1996, 1997–2007, and 2008–2015, respectively. The HIV-incidence rate among Israeli MSM slightly declined from 2012, after peaking in 2011 at 6.2 per 100,000.

**Conclusions:**

The recent reduction in HIV-incidence and in AIDS diagnoses among Israeli MSM is encouraging. Nevertheless, the disproportionate incidence of HIV among MSM requires sustained efforts to abate further infections.

## Background

The HIV epidemic in Israel, one of the 53 WHO European region countries, mostly affects specific groups, similarly to other low HIV prevalence countries [1–6]. The major risk categories in Israel are: 1) people living with HIV (PLWHIV) originating from country with Generalized HIV Epidemic (OGE); 2) Men who have sex with men (MSM); and 3) Intravenous Drug Users (IDU) [4–6]. The prevalence of these risk groups changes throughout the study period, most notably with mass immigration from countries with a generalized HIV epidemic in the 1990s and ongoing undocumented migration. MSM accounted for over 30% of all new HIV infections and 46% of new HIV cases among men notified to the Israeli Ministry of Health (MoH) between 2011 and 2015. HIV incidence rates among MSM in Israel are comparatively lower than reported in other developed countries and have recently begun to decline [4]. In 2015, the incidence rate of HIV among MSM in Israel, Western Europe and the United States was 4.9, 9.9 and 24.9 cases per 100,000 men, respectively [1, 2, 4].

Antiretroviral therapy (ART) became available under Israel’s universal National health insurance in 1997, in the form of nucleoside-analog reverse transcriptase inhibitors and the introduction of the triple drug combinations utilizing protease inhibitors. These drugs often caused several side-effects, which in times deterred patients" compliance and therefore did not inhibit progress to acquired immunodeficiency syndrome (AIDS) as much as expected [7]. As of 2008, HAART (Highly Active Anti-Retroviral Therapy), including integrase inhibitors with significantly less side effects, were introduced. Available ART currently includes nucleoside analogue reverse transcriptase inhibitor, non-nucleoside reverse transcriptase inhibitors, protease inhibitors, integrase inhibitors, CCR5 receptor inhibitors and maturation inhibitors, and is fully subsidized by the National Israeli health insurance to all HIV-infected persons who are clinically followed in the HIV treatment centers. The aim of this study is to describe HIV epidemiological trends and clinical characteristics of MSM living with HIV in Israel since the first report in 1981.

## Methods

This retrospective study includes all individuals reported to the Israeli MoH with HIV and self-identified as MSM between 1981 and 2015. MSM also reported as being IDU were separately analyzed. HIV and clinical information, including development of the AIDS defining diseases and mortality are notified to the National HIV/AIDS Registry (NHAR) operated by the Department of Tuberculosis and AIDS (DTA) at the MoH. The reports are sent by different health departments and HIV treatment centers following patient interviews that include personal identifiers, behavioral characteristics and additional clinical information.

HIV tests in Israel are free of charge at primary health care clinics, HIV treatment centers, and sexually transmitted infection (STI) clinics to both citizens who present with a National Israeli identity document and to non-citizens who present with a passport whether tourists, students, workers, or migrants. Data were analyzed separately for citizens and non-citizens due to different demographic determinants. Most tests in Israel are performed confidentially, where only medical personnel involved has access to the identity of the patient, Anonymous tests are also available, where nobody knows the identity of the patient, who is given a random number. Peripheral laboratories send their positive enzyme-linked immunosorbent assay results to the National HIV Reference Laboratory (NHRL) which performs a confirmatory Western blot assay, genetic profiling, and the majority of drug resistance analyses, reporting its findings to the MoH.

AIDS defining diseases were characterized according to the 1993 European AIDS surveillance case definition and the updated versions [8]. AIDS-related deaths were identified by cross-referencing deaths of persons from the NHAR to their medical files and death certificates. Rate calculations were performed using the population size for all Israeli men aged 15–64 at any notifying year published by the Israeli Central Bureau of Statistics [9]. Interquartile ranges for each year of notification were generated for age at HIV and AIDS notification. Survival analyses for years between HIV diagnosis to AIDS diagnosis, and between HIV diagnosis and death were performed using the Kaplan-Meier (KM) method and the Mantel-Cox log-rank test presented by 95% confidence intervals. KM estimates were generated for three different HIV periods: 1981–1996, 1997–2007, and 2008–2015, used as proxies for different treatment regimen available in Israel during each period. The first period (pre-antiretroviral therapy – pre-ART) encompasses when HIV was diagnosed but lacked treatment options [10]. The second period (early-ART) includes the availability of first generation ART in Israel, specifically nucleoside-analog reverse transcriptase inhibitors and the introduction of the triple drug combinations utilizing protease inhibitors [11, 12]. The third period (late-ART) began in 2008, when integrase inhibitors became available with fewer adverse drug reactions than previous treatment regimens [13]. AIDS-defining diseases were analyzed by period of AIDS diagnosis and total disease diagnosis and percentages were calculated.

Power analysis was not required as it includes the entire population rather than a sample. When calculating the cumulative survival probability between HIV notification and AIDS diagnosis, survival was calculated as years elapsing from date of HIV diagnosis until the date of an AIDS diagnosis. Censored cases included all cases that either died or left Israel since being diagnosed, or were never diagnosed with AIDS. In cumulative survival probability between HIV notification and all-cause mortality, survival was calculated as the time elapsed from date of HIV diagnosis until the date of death. All cases that died of all causes were included and those who had left the country or lived by the end of 2015 were censored. A second set of survival analysis was conducted among HIV-infected patients until AIDS-related death, where censored cases included those who had died from all other causes, had left Israel or lived beyond the end of 2015. In survival analysis among AIDS patients to death, censored cases included those who left Israel or lived beyond the end of 2015.

Survival was further analyzed using Cox’s proportional hazard analysis to examine covariates associated with survival among age at HIV diagnosis and the period of HIV diagnosis. First, each aforementioned period-group was analyzed separately and then age was entered in a stepwise conditional proportional-hazard regression model. The latest period (2008–2015) was used as the reference group during analysis and was iterated 20 times.

All statistical analyses were performed using SPSS software (V.20.0) (Statistical Package for Windows, SPSS Inc., Chicago IL, USA).

This data analysis included information retrieved for surveillance purposes (mandatory by Israeli law) and therefore the need for Helsinki ethical review board approval was not required. The corresponding author is the Director of the DTA, and the material could be obtained from him if the request is in accordance to the Israeli MoH requirements.

## Results

Of all 8850 people living with HIV (PLWHIV) cases ever reported to the NHAR between 1981 and 2015, 2151 (24.3%) were self-identified as MSM (of those, 2052 [95.4%] were Israeli citizens and 99 [4.6%] were non-Israeli citizens). MSM represented 41.5% of all male cases among Israeli citizens. AIDS was diagnosed in 296 PLWHIV Israeli MSM, of whom 154 (52%) were “surprise” cases – in other words, notified with AIDS diagnosis before they were aware they had HIV-infection. Eighteen AIDS were diagnosed among non-Israeli patients, of whom 14 were surprise cases. By the end of 2015, of the 2052 MSM Israeli citizens infected with HIV, 1747 (85.1%) were living in Israel, 44 (2.1%) have left the country, and 261 (12.7%) died--of those, 137 (52.5%) were AIDS-related deaths.

**Fig. 1:**
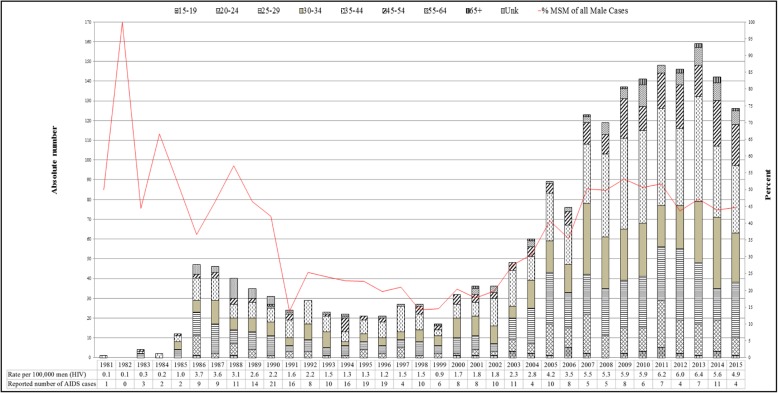
Newly-Diagnosed HIV Cases among MSM* Israeli citizens, and their proportion out of all male HIV cases, Israel, 1981–2015. *MSM – Men Having Sex with Men

As shown in Fig. 1, the overall rates of HIV increased during the study period, up to a peak of 6.2 per 100,000 men in 2011. Since then, a gradual but fluctuating decline was shown from 6.2 to 4.9 cases per 100,000 men by the end of 2011 and 2015, respectively.

**Fig. 2:**
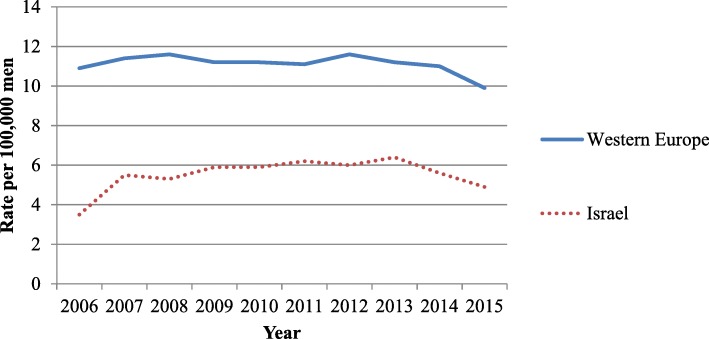
Newly-diagnosed HIV cases among MSM* per 100,000 men, 2006–2015. *MSM – Men having Sex with Men

The trend profile in rates of new HIV diagnoses found among the MSM population are similar to those found in Western European populations from 2006 until 2015 [1]: after increasing rates, a plateau was observed during 2007 until approximately 2012, followed by a recent decrease (Fig. 2).

**Fig. 3:**
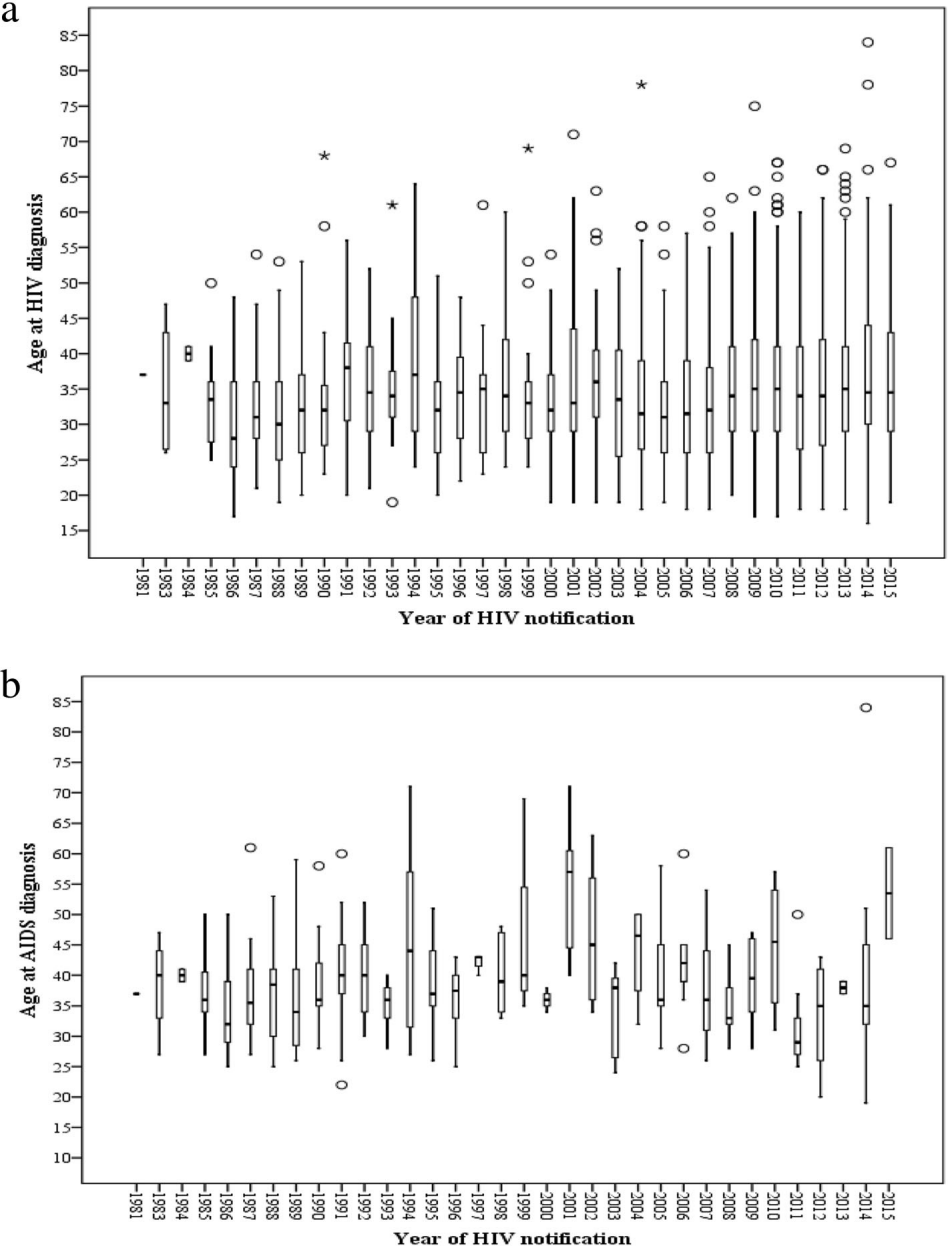
IQR at notification age, by year of (**a**) HIV diagnosis and (**b**) AIDS diagnosis, Israel 1981–2015

The mean age at notification for the entire study period among Israelis was 34.9 ± 9.96 years (median 33, range 16–84). Among all age groups, 25–29 and 30–34 year-olds experienced the highest risk of infection. Only 4 cases that were not vertically infected were ever reported to be under the age of 18. From 1981 to 1996, the mean age at HIV diagnosis was 33.5 ± 8.5 (*N* = 333). The mean age at AIDS diagnosis was 37.7 ± 8.4 (*N* = 186). From 1997 to 2007, the mean age at HIV diagnosis was 33.7 ± 9.6 (*N* = 571) and the mean age at AIDS diagnosis was 41.9 ± 10.3 (*N* = 69). From 2008 to 2015 the mean age at HIV diagnosis was 35.9 ± 10.4 (*N* = 1118) and at AIDS diagnosis was 38.2 ± 12.0 (*N* = 41). While the absolute caseload has increased, the proportion of notifications from each age group has remained steady. Interquartile range (IQR) models illustrated a steady median age at notification from 1981 to 2015; for the aforementioned periods the median ages at diagnosis were 32 (IQR = 12), 32 (IQR = 12) and 34 (IQR = 14), respectively (Fig. 3). Similar patterns were also observed for age at AIDS notification (not shown).

### AIDS defining diseases by period of treatment availability

The complete description of AIDS-defining diseases by period of available treatment is presented in Table 1. Due to the occurrences of multiple diagnoses per case, percentages can exceed 100%.

**Table 1:** AIDS-defining diseases among Israeli Men having sex with Men according to HIV treatment regimen period, Israel, 1981–2015

HIV treatment regimen period	1981–1996		1997–2007		2008–2015		1981–2015	
Number of AIDS cases	*N* = 186		*N* = 69		*N* = 41		*N* = 296	
Diseases	Diagnosis	%^a^	Diagnosis	%^a^	Diagnosis	%^a^	Diagnosis	%^a^
*Pneumocystis carinii* pneumonia	81	43.5	19	27.5	18	43.9	118	39.9
Kaposi’s sarcoma	44	23.7	10	14.5	5	12.2	59	19.9
Wasting syndrome due to HIV	32	17.2	16	23.2	1	2.4	49	16.6
Candidiasis, esophageal	22	11.8	6	8.7	2	4.9	30	10.1
Toxoplasmosis of brain in a patient over one month of age	22	11.8	4	5.8	1	2.4	27	9.1
Cytomegalovirus retinitis	21	11.3	0	0.0	0	0.0	21	7.1
Cryptococcosis, extrapulmonary	15	8.1	1	1.4	3	7.3	19	6.4
Opportunistic Infections, not specified	9	4.8	6	8.7	4	9.8	19	6.4
*Mycobacterium avium* complex or M. *kansasii*, disseminated or extrapulmonary	14	7.5	1	1.4	1	2.4	16	5.4
Pneumonia, recurrent in an adult or an adolescent (aged 13 years or over)	11	5.9	2	2.9	2	4.9	15	5.1
Cytomegalovirus disease (other than liver, spleen or nodes) in a patient over one month of age	9	4.8	3	4.3	2	4.9	14	4.7
Lymphoma, not specified	9	4.8	3	4.3	1	2.4	13	4.4
Cryptosporidiosis, intestinal with diarrhea (> 1 month duration)	9	4.8	2	2.9	0	0.0	11	3.7
Lymphoma, immunoblastic	5	2.7	5	7.2	0	0.0	10	3.4
Lymphoma, Burkitt’s	2	1.1	3	4.3	4	9.8	9	3.0
Herpes simplex: chronic ulcer(s) (> 1 month duration) or bronchitis, pneumitis or esophagitis in a patient over 1 month of age	8	4.3	0	0.0	0	0.0	8	2.7
Lymphoma, primary, of the brain	6	3.2	0	0.0	1	2.4	7	2.4
Encephalopathy, HIV-related	5	2.7	1	1.4	1	2.4	7	2.4
Progressive multifocal leukoencephalopathy	4	2.2	2	2.9	0	0.0	6	2.0
*Mycobacterium tuberculosis*, pulmonary in an adult or adolescent (aged 13 or over)	0	0.0	4	5.8	2	4.9	6	2.0
*Mycobacterium tuberculosis*, pulmonary in an adult or adolescent	1	0.5	2	2.9	1	2.4	4	1.4
*Mycobacterium*, other species or unidentified species, disseminated or extrapulmonary	2	1.1	0	0.0	0	0.0	2	0.7
Salmonella (non-typhoid) septicaemia, recurrent	0	0.0	2	2.9	0	0.0	2	0.7
Candidas of the bronchi, trachea or lungs	1	0.5	0	0.0	0	0.0	1	0.3
Histoplasmosis, disseminated or extrapulmonary	1	0.5	0	0.0	0	0.0	1	0.3
Total number of diagnoses	333	179.0	92	133.3	49	119.5	474	160.1

(^a^) The percentage can exceed 100% due to the fact that there are sometimes multiple AIDS defining diseases per case

From 1981 to 1996, 333 diagnoses of AIDS-defining diseases were made among 186 AIDS patients (51.2% of all HIV/AIDS MSM diagnosed during this period). From 1997 to 2007, of 69 patients diagnosed with AIDS (12.1% of all HIV/AIDS MSM diagnosed during this period) 92 AIDS-defining disease diagnoses were made. Between 2008 and 2015, of 41 AIDS patients (3.7% of all HIV/AIDS MSM diagnosed during this period), 49 AIDS-defining diseases were diagnosed. During the three respective periods, *Pneumocystis carinii* pneumonia (PCP) (39.9%), Kaposi’s sarcoma (KS) (19.9%), and wasting syndrome (WS) (16.5%) were consistently the three most common diseases among Israeli MSM who had developed AIDS.

In terms of overall prevalence, PCP dropped from 435.5 to 275.4 between 1997 and 2007 but rose up to 439 per 1000 MSM AIDS cases during the subsequent period. The prevalence of KS among MSM diagnosed with AIDS steadily declined over the periods (from 236.6 per 1000 MSM AIDS cases during the first period, to 144.9 during the second, to 122 per 1000 MSM AIDS cases during the third). The prevalence of WS declined as well from 172 per 1000 MSM AIDS cases during the first period, subsequently rising to 231.9 during the second, then plummeting to 24.4 per 1000 MSM AIDS cases.

### MSM reported as also being intravenous drug users (IDU)

Among the 38 MSM IDU reported as HIV positive to the NHAR between 1981 and 2015, 16 developed AIDS. At the end of 2015, 21 were living in Israel, 16 had died, and 1 had left the country. Only one MSM IDU case was not of Israeli citizenship. During the three studied periods, 24, 6 and 9 AIDS diagnoses were made, respectively, among MSM IDU. Altogether (in term of diagnosis), during the first period, 17 AIDS-defining diseases (81%) were diagnosed. in the second 2 AIDS-defining diseases (9.5%) were diagnosed, and in the third period 2 (9.5%) were diagnosed. The three most common AIDS-defining diseases in all three periods were PCP (4 diagnoses), toxoplasmosis of the brain (4 diagnoses), and wasting syndrome (4 diagnoses).

### Time from HIV notification to AIDS diagnosis

**Fig. 4:**
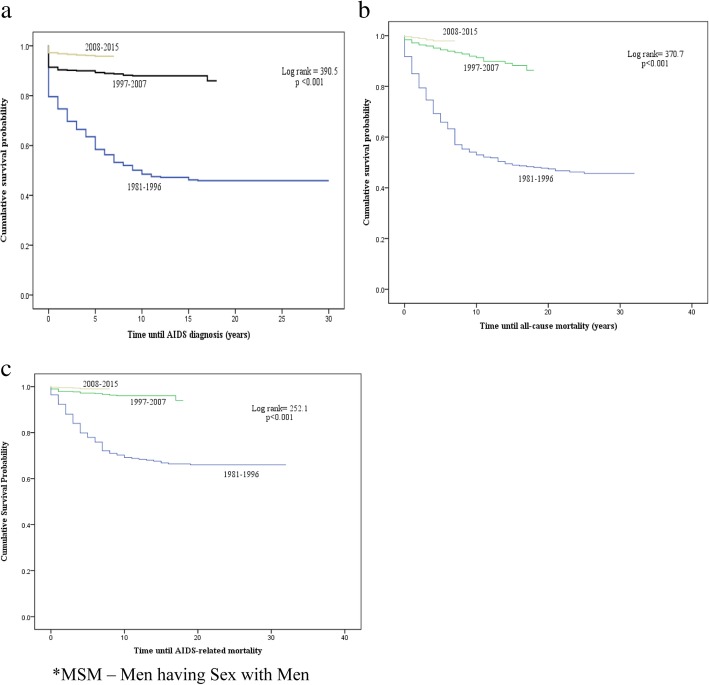
Cumulative survival probabilities of (**a**) AIDS diagnosis (**b**) all-cause mortality(**c**) and AIDS-related mortality, among Israeli MSM* patients diagnosed with HIV, according to treatment periods

During the three time periods previously mentioned, the total numbers of newly reported HIV cases and of AIDS diagnosis were as follows: 363, 571 and 1118 for HIV alone, and 186, 69 and 41 for AIDS (Table 1), respectively. The average time between HIV notification and AIDS diagnosis in 1981–1996, 1997–2007, and 2008–2015 were 15.5 (95% CI 14.0–16.9), 16.0 (95% CI, 15.5–16.4), and 6.7 (95% CI, 6.7–6.8) years, respectively. Improvements in reduced AIDS diagnoses among patients from the second and especially the last period compared to the first period is evident (log-rank tests *p* < 0.0001, Fig. 4a). In the Cox regression analysis, the risk of an AIDS diagnosis was greater for both periods 1981–1996 (HR: 14.1; 95% CI: 10.0–19.9; *p* < 0.001) and 1997–2007 (HR: 2.4; 95% CI: 1.7–3.7; *p* < 0.001) compared to the last period 2008–2015. When controlling for age, the risk of an AIDS diagnosis was HR: 1.03 (95% CI: 1.0,-1.0; *p* < 0.001).

### Time from HIV notification to death of all causes

During the three intervals studied, 190 (1981–1996), 56 (1997–2007), and 17 (2008–2015) HIV-infected MSM died representing 52.3, 9.8, and 1.5% of HIV-infected MSM, respectively. The overall average time between HIV diagnosis and death among those who died was 25.9 (95% CI, 25.1–26.6) years. The average estimated time from HIV/AIDS notification to death in 1981–1996, 1997–2007, and 2008–2015 was 17.5 (95% CI, 16.0–18.9), 16.6 (95% CI, 16.2–17.0) and 6.9 (95% CI, 6.8–6.9) years, respectively. A sharp decrease in mortality between the latter two groups and the earliest period is apparent, log-rank *p* < 0.0001 (Fig. 4b). However, that discrepancy could be explained by the fact that time elapsed between the last study period and the present day is far below the average for the other two periods. The risk of all-cause mortality among MSM diagnosed with HIV was greater for both periods 1981–1996 (HR: 21.4; 95% CI: 12.9–35.4; *p* < 0.001) and 1997–2007 (HR: 3.1; 95% CI: 1.8–5.4; *p* < 0.001) compared to the last period 2008–2015. When controlling for age, the risk of death was HR: 1.0 (95% CI: 1.0–1.0; *p* < 0.001).

### Time from HIV notification to AIDS-related mortality

During the study period, the reported number of AIDS-related deaths was 106, 23 and 8 for each period, respectively. The average estimated time from HIV/AIDS notification to AIDS-related death in 1981–1996, 1997–2007, and 2008–2015 was 22.7 (95% CI, 21.3–24.2), 17.4 (95% CI, 17.1–17.6) and 7.9 (95% CI, 7.9–8.0) years, respectively. The overall average of time between HIV diagnosis and AIDS-related death was 28.9 (28.3–29.4) years. Death among the latter two groups was similar, while the earliest period demonstrated higher rates, log-rank test *p* < 0.0001 (Fig. 4c).

### Time from AIDS diagnosis to all-cause mortality

The total number of deaths among MSM diagnosed with AIDS during the three periods was 161, 29 and 12 deaths among 186, 69 and 41 AIDS patients, respectively. The mean time from AIDS diagnosis until death in years was 5.1 (95% CI, 3.8–6.3), 10.3 (95% CI, 8.5–12.2) and 5.0 (95% CI, 4.0–5.9), log-rank test *p* < 0.0001.

## Discussion

MSM remains the second most common route of HIV transmission in Israel, and the most common among men and Israeli-born. We can observe that similarly to the Israeli population in which the proportion of Israeli born has increased from 35% in 1948 to 50% in 2000 to 75% in 2016, the proportion of Israeli born MSM living with HIV increased with time [14]. Diagnosis rate among MSM has steadily increased between 1981 and 2011, hit a peak, and has since been on the decline. This decline could be primarily be attributed to the expanded availability of ART that has become easier to take and safer, in addition to a reduction in risky sexual behaviours for a certain proportion of the MSM community, due to increased education and reduced stigma in recent years.

Despite expanded ART therapy and the recent declines, the MSM population continues to experience a disproportionate HIV burden, accounting for over a quarter of all HIV cases in Israel while only estimated to constitute some 3% of the male population [15]. Individual-level risks for HIV acquisition include unprotected anal intercourse, high number of male sex partners and concurrency, and recreational drug use before or during sex [16, 17]. A phylogenetic analysis of subtype B virus, more common among MSM, was performed in Israel and found an increasing number of clusters [18] were co-infected with syphilis [19]. This suggests the existence of unprotected anal sex and sexual networks characterized by high-risk behavior. Community-level risks include higher numbers of sexual partners facilitated by use of social networking websites and apps, and acceptability of partner fluidity [20].

While MSM in Israel are disproportionately affected by HIV, the burden is lower than in other developed countries--the average Israeli HIV infection rate is half of the average Western Europe rate: 4.9 per 100,000 men compared to 9.9 per 100,000 men, respectively [1]. The difference between Israeli and European rates may be due to a combination of cultural determinants. Israel has characteristically high rates of male circumcision (MC), where nearly all males are circumcised according to Jewish or Islamic tradition [15]. This may be a potential factor in the observed lower rates where MC has been proven to significantly decrease HIV incidence among heterosexual males [21] although conclusive evidence pointing to a similar effect among MSM is lacking [3, 22]. However, an ecological study found HIV incidence in Israel among heterosexuals to be lower than other similar developed countries in all aspects except MC [23]. Further research is required to determine the protective effect of MC among Israeli MSM.

The significant improvement in survival outcomes between the three periods are influenced by the protective impact of ART in people living with HIV/AIDS, both in delaying the development of AIDS and the risk of death, as reported in international reports [24, 25]. However, for the last period, it should be highlighted that the period is truncated when compared to the other periods. Additionally, the middle period could be considered as a truncated period to a lesser degree. In addition, during the last period, advances in ART regimens, including a better safety profile and fewer daily dose regimens has increased drug adherence in comparison to earlier periods [26, 27]. Preventative measures including pre-exposure prophylaxis (PrEP) were not yet available during the study period, but are offered as of September 2017.

Interestingly, while the incidence of HIV has increased, the proportion of notifications from each age group remained steady. Although information on the precise time of infection may not be determined, the stable median age supports the validity of the results, showing no significant changes over time, even as testing has become more accessible along with the study periods. The pattern observed in our cohort is akin to the pattern observed in median ages in Norway [28]. The few HIV cases under the age of 18 are an important characteristic of the Israeli MSM HIV/AIDS population. While it is common for younger MSM to undergo routine HIV testing less frequently than their older counterparts [29], the rate of young MSM under the age of 18 notifying infection is much smaller than rates observed in other countries, such as the United States [30].

The three most common AIDS-defining diseases found among the Israeli MSM cohort are similar to findings from Western Europe, Canada, and the US, where the three common defining diseases in 2015 were *Pneumocystis carinii* Pneumonia, oesophageal candidiasis and Kaposi’s sarcoma [1, 31].

This study is subject to several limitations. First, the lack of CD4 count/Viral Load (VL) and ART treatment records at the NHAR limited our ability to conduct more detailed analyses. Second, MSM classification was based self-reporting, which may prove unreliable due to the stigma or taboo of homosexuality among some of the more conservative and religious social strata [15]. Sexual behavior is a sensitive matter and is subject to reporting bias, which may have resulted in a misclassification of MSM as heterosexuals, and a consequent underestimation of PLWHIV that are part of the MSM risk group. If occurred, this underestimation is non-differential. An additional limitation resides in the unequal years captured in each period. While the periods reflect treatment regimens available in Israel in each period, the differences in the number of cases may slightly contribute to a biased comparison. Last, AIDS-related mortality was used to give a more ‘accurate’ picture of survival estimates among HIV-positive MSM than just all-cause mortality. However, national medical records, and specifically cause of death, are subject to potential misclassification.

## Conclusion

This is the first Israeli national epidemiological study on the HIV epidemic among MSM living with HIV/AIDS. This study’s strength relies on the updated and monitored NHAR, and the capability of cross-referencing with the Civil Registry for deaths and those who left Israel. The observed decline in HIV rates in recent years and improved survival outcomes are encouraging. The proliferation and advances in ART have significantly decreased HIV/AIDS-related morbidity and all-cause mortality, ultimately improving the quality of life of people living with HIV. However, HIV infection incidence still remains disproportionately high among the MSM population, where therapy adherence failure and loss to follow-up pose serious challenges in reducing HIV infections. The Israeli MoH already offers free access to the health care services and facilitates interventions to relieve the burden of HIV/AIDS in the MSM community in joint partnerships with local non-governmental organizations and continues to reduce the burden of HIV in Israel. Sustained efforts to abate HIV infection and AIDS development in this vulnerable community are still necessary.

## Data Availability

Data is not publically available. Material could be obtained from the corresponding author if the request is in accordance to the Israeli Ministry of Health requirements.

## Abbreviations

AIDS: Acquired Immune Deficiency Syndrome
ART, HAART: Antiretroviral Therapy, Highly Active Antiretroviral Therapy
DTA: Department of Tuberculosis and AIDS
HIV: Human Immunodeficiency Virus
HR: Hazard Ratio
IDU: Intravenous Drug Users
IQR: Interquartile range
KM: Kaplan-Meier
KS: Kaposi’s Sarcoma
MC: Male Circumcision
MoH: Ministry of Health
MSM: Men who have Sex with Men
NHAR: National HIV/AIDS Registry
NHRL: National HIV Reference Laboratory
OGE: Originating from country with Generalized HIV Epidemic
PCP: *Pneumocystis Carinii* Pneumonia
PLWHIV: People living with HIV
PrEP: Pre-Exposure Prophylaxis
STI: Sexually Transmitted Infection
WS: Wasting Syndrome
